# First-in-Human PET Imaging of [^18^F]SDM-4MP3: A Cautionary Tale

**DOI:** 10.1155/2023/8826977

**Published:** 2023-09-08

**Authors:** Kimberly L. Desmond, Anton Lindberg, Armando Garcia, Junchao Tong, Michael B. Harkness, Elena Dobrota, Kelly Smart, Carme Uribe, Jeffrey H. Meyer, Sylvain Houle, Antonio P. Strafella, Songye Li, Yiyun Huang, Neil Vasdev

**Affiliations:** ^1^Azrieli Centre for Neuro-Radiochemistry & Brain Health Imaging Centre, Centre for Addiction and Mental Health (CAMH), Toronto, Ontario, Canada; ^2^Department of Psychiatry, University of Toronto, Ontario, Canada; ^3^Krembil Brain Institute, University Health Network, University of Toronto, Ontario, Canada; ^4^PET Center, Department of Radiology and Biomedical Imaging, Yale University, New Haven, Connecticut, USA

## Abstract

[^18^F]SynVesT-1 is a PET radiopharmaceutical that binds to the synaptic vesicle protein 2A (SV2A) and serves as a biomarker of synaptic density with widespread clinical research applications in psychiatry and neurodegeneration. The initial goal of this study was to concurrently conduct PET imaging studies with [^18^F]SynVesT-1 at our laboratories. However, the data in the first two human PET studies had anomalous biodistribution despite the injected product meeting all specifications during the prerelease quality control protocols. Further investigation, including imaging in rats as well as proton and carbon 2D-NMR spectroscopic studies, led to the discovery that a derivative of the precursor had been received from the manufacturer. Hence, we report our investigation and the first-in-human study of [^18^F]SDM-4MP3, a structural variant of [^18^F]SynVesT-1, which does not have the requisite characteristics as a PET radiopharmaceutical for imaging SV2A in the central nervous system.

## 1. Introduction

[^18^F]SynVesT-1 (a.k.a. [^18^F]SDM-8 or [^18^F]MNI-1126) is a PET radiopharmaceutical that binds to the synaptic vesicle protein 2A (SV2A) and is considered to be a biomarker of synaptic density [1]. This target is ubiquitous throughout neuron-populated brain regions [2], and studies using a carbon-11 labeled derivative, [^11^C]UCB-J, have found reduced SV2A binding across diverse disorders including depression [3], Alzheimer's disease [4], cannabis use disorder [5], schizophrenia [6], and Parkinson's disease [7]. First-in-human studies of [^18^F]SynVesT-1 conducted at the Yale PET Center yielded excellent brain volume of distribution (*V*_*T*_) in the cortical and subcortical regions [8]. Blocking studies with levetiracetam [9] revealed minimal off-target binding of [^18^F]SynVesT-1, and the radiopharmaceutical is a promising agent for noninvasive PET imaging (SUVR, SRTM [10]) using the central white matter (centrum semiovale) with low specific uptake as a reference region [11].

To our surprise, a concurrent human pilot study conducted at the Centre for Addiction and Mental Health (CAMH) did not yield comparable results to the [^18^F]SynVesT-1 PET imaging studies carried out at the Yale PET Center (*vide supra*). Here, we report a subsequent investigation of the radiosynthesis and human imaging pipeline, supported by rodent imaging experiments, from which we concluded that a 4-methylpyridin-3yl precursor material had been provided to CAMH from a commercial supplier, instead of the expected 3-methylpyridin-4yl-based precursor. The structural differences between the radiolabeling precursors were subtle enough for the resulting new radiopharmaceutical, [^18^F]SDM-4MP3, to pass the routine quality control protocols, and it was not metabolized in a significantly different way from [^18^F]SynVesT-1. However, major differences were observed upon initial human brain PET imaging studies. Once the erroneous precursor was identified, pilot scans were undertaken in two additional participants using product synthesized from the correct precursor, producing results consistent with the original observations from PET imaging with [^18^F]SynVesT-1 [8].

## 2. Materials and Methods

### 2.1. Radiotracer Synthesis

The [^18^F]SDM-4MP3 radiolabeling precursor, identified as SDM-8 tin precursor according to the Certificate of Analysis (CoA) (product no. 3425.0002.500, manufactured June 2019), was obtained from ABX Advanced Biochemical Compounds GmbH (Radeberg, Germany). Radiopharmaceutical synthesis was performed on-site at CAMH according to the published method for [^18^F]SynVesT-1 [12]. Coinjections of radiolabeled product [^18^F]SDM-4MP3 with SynVesT-1 reference standard on reverse phase HPLC (Luna C18 10 *μ*m 250 × 10 mm column, 30 : 70 acetonitrile-to-water (0.1 N ammonium formate + 0.5%acetic acid), 5 mL/min) were performed to verify product identity and radiochemical purity. Enantiomeric purity was confirmed by chiral analytical HPLC (Lux 5 *μ*m cellulose-1 150 × 4.6 mm column using 40 : 60 acetonitrile-to-water (0.1 N ammonium formate + 0.5% formic acid at 1 mL/min) by coinjecting the radiolabeled product with a mixture of (*R*)- and (*S*)-SynVesT-1 reference standard obtained from the Yale PET Center. The radiopharmaceutical was formulated in a sterile, pyrogen-free isotonic saline solution as required for *in vivo* PET imaging studies. For later comparison, precursor testing and synthesis were performed identically with the [^18^F]SynVesT-1 precursor obtained from the Yale PET Center (New Haven, CT, USA) and PharmaSynth AS (Tartu, Estonia).

### 2.2. Human Brain PET Studies

All human components of the study were approved according to the Research Ethics Board for Human Subjects at CAMH (CTA 125, REB 075/2019). All participants were recruited from the community and provided written informed consent. Participant inclusion criteria were self-report of good health. Exclusion criteria included any history of neurological or psychiatric disorder; cognitive impairment according to MoCA assessment (score less than 26); pregnancy and/or nursing; and disorders of coagulation, blood, or ongoing use of anticoagulant medication. Each participant's height, weight, current medications, and smoking status was recorded. Participants abstained from taking dopaminergic medications, including aspirin [13], for 24 hours prior to the PET exam (see Table 1).

#### 2.2.1. PET Imaging

Participants were scanned on a 5-ring GE Discovery MI PET/CT (GE Healthcare Technologies, Chicago, Illinois). Participants 1 and 2 (P1 and P2) were scanned with [^18^F]SDM-4MP3 (the radiopharmaceutical synthesized from the ABX precursor), and [^18^F]SynVesT-1 (synthesized from the Yale precursor) was used for PET imaging of participants 3 and 4 (P3 and P4). A thermoplastic facemask (Tru-Scan Imaging, Annapolis) was used to restrict head motion within the PET scanner bore. CT was used to acquire a scout scan, followed by a low-dose scan for attenuation correction. The radiopharmaceutical (target dose 185 MBq ± 10%, molar activity > 11 GBq/*μ*mol) was delivered starting 30 s after the initiation of the emission scan, with a smooth bolus injection by hand (2-5 s) via intravenous line placed in the antecubital vein, followed by a 10 mL saline flush to clear residual activity from the line. Emission data was acquired for 120-125 min, allowing up to 5 min for a background frame sampled at 1 s intervals to determine the precise starting time of brain uptake, and used to establish the duration of the background frame inserted prior to dynamic frame reconstruction. Data acquired after the start of brain uptake was reconstructed into frames using FORE rebinning and filtered back-projection, using a Hanning filter at the Nyquist frequency to create a dynamic image set. Frame definitions were 30 s × 3, 60 s × 3, 120 s × 2, 300 s × 22 (total = 120 min). For use as a reference for motion correction, images were also reconstructed without attenuation correction using an iterative method with 4 subsets and 6 iterations. All reconstructions were performed using vendor algorithms on the GE DMI console. Reconstructed images were 256 × 256 × 89 voxels and had a field of view of 400 mm in-plane and 250 mm axially, corresponding to a voxel dimension of 1.5625 × 1.5625 × 2.79 mm. A spatial resolution of approximately 5.8 × 5.8 × 5.6 mm was achieved (measured in accordance with National Electrical Manufacturers Association NU 2-2007 standards). The mean injected activity was 192.7 MBq for [^18^F]SDM-4MP3 and 184.3 MBq for [^18^F]SynVesT-1 (Table 1).

#### 2.2.2. Arterial Input Function Measurement

The arterial line was placed into the radial artery by a qualified respiratory therapist. Up to 160 mL of arterial blood was collected via automated blood sampler (ABSS, Comecer PBS-101) and via manual samples for plasma metabolite correction. The ABSS protocol was started ~15 s after emission scan start, and pump speed was set to 300 mL/h, to reduce dispersion in ABSS lines during maximum rate of change of arterial activity concentration, at 1 s sampling period for 5 min and 2 s sampling period for 136 s. Pump speed was decreased to 180 mL/h at a sampling period of 5 s for the following 360 s and a sampling period of 10 s for 540 s, for a total sampling time of 22 min and 16 s. Manual samples were collected at baseline, 2.5, 7, 12, 15, 20, 30, 45, 60, 90, and 110 min relative to emission scan start into PTFE tubes, followed by a saline flush for all except 2.5- and 7-min samples to avoid discontinuities in arterial data during peak uptake.

Baseline whole blood samples were centrifuged at 3100 RPM at room temperature for 5 min to extract plasma. Plasma supernatant was centrifuged for 20 min at 1100G at 22°C to extract filtrate for protein binding analysis. Each subsequent manual sample was centrifuged at 3900 rpm at 4°C for 5 min, and 0.3 mL of whole blood and 0.3 mL of plasma were extracted and measured on a gamma counter (Wizard 2480, Perkin Elmer, Turku, Finland) to quantify activity concentration.

*(1) Column-Switching HPLC*. Plasma analysis was adapted from a column-switching HPLC method developed by Luthra and Hilton and coworkers [14, 15], as previously described [16]. To separate parent compound from radiometabolites, plasma was treated with urea and centrifuged (3900 rpm, 4°C, 5 min) under experimental conditions described for [^11^C]UCB-J [17]. Samples were initially analyzed in capture mode with the capture solvent flow rate of 1.8 mL/min for 4 min before switching to analysis mode with the analysis solvent running at 1.8 mL/min for an additional 12 min. The metabolite-corrected arterial input function was calculated as the product of the dispersion-corrected, merged (automatic and manual), and smoothed plasma activity curve and the smoothed parent fraction curve using in-house software [18, 19].

#### 2.2.3. Magnetic Resonance Imaging

A routine T1-weighted anatomical scan (BRAVO sequence, GE Healthcare, 3T Discovery MR750) was acquired for anatomical coregistration as part of a 1-hour MRI protocol. Resolution was 0.9 mm isotropic; TE/TR = 3.00 ms/6.74 ms; TI = 650 ms; flip angle = 8°; scan time: 4 min and 42 s.

#### 2.2.4. PET Image Processing

*(1) Image Registration and Region of Interest*. PET images were registered to the subject's corresponding *T*_1_-weighted anatomical MRI and time-activity curves (TACs) extracted for regions of interest (ROIs) defined in the Hammers-N30R83 atlas in MNI space using the PNEURO module in Pmod 4.2 (Pmod Technologies LLC, Switzerland). The centrum semiovale (CS) reference region was merged from a separate ROI, CS-AAL, provided by Rossano et al. [11].

*(2) Quantitative Analysis*. Regional standard uptake value ratio (SUVR) for each ROI was computed by measuring the frame-duration weighted average TACs between 60 and 90 min, divided by the value obtained for the CS. SUVR images were created from frame-duration weighted average activity between 60 and 90 min, the optimal window investigated in Naganawa et al. [8], using centrum semiovale as a reference region [11]. Regional volume of distribution (*V*_*T*_) was estimated from 1-TCM using the calculated arterial input function and the PKIN module in Pmod 4.2 for kinetic modelling.

### 2.3. Preclinical PET Imaging Studies

Preclinical PET imaging studies were approved by the Animal Care Committee at CAMH (Animal Use Protocol #838). Six adult Sprague-Dawley rats were scanned for 120 min on either a Mediso nanoScan PET/MR 3 T or PET/CT scanner (Budapest, Hungary), and the images were analyzed using VivoQuant® software (version 4.1, Invicro LLC, Needham, MA, USA), as described previously [20]. Five rats (3 M/2F, 528 ± 46 g) were used for the baseline [^18^F]SynVesT-1 scans (injected dose: 13.3 ± 3.2 MBq; molar activity: 157 ± 64 GBq/*μ*mol; mass injected: 0.22 ± 0.08 nmol/kg), with two of the male rats also scanned after injection of the blocking drug levetiracetam (cat#L1784, LKT Laboratories Inc, St Paul, MN, USA) at 30 mg/kg (i.v., 15 min before the tracer; injected dose: 10.2 ± 0.1 MBq; molar activity: 78 ± 15 GBq/*μ*mol; injected mass: 0.24 ± 0.04 nmol/kg). One female rat (273 g) was used for the 120 min scan with [^18^F]SDM-4MP3 (injected dose: 13.0 MBq; molar activity: 125.5 GBq/*μ*mol; injected mass: 0.38 nmol/kg). Acquired list mode data were sorted into thirty-nine 3-dimensional (3D) (3 × 5 s, 3 × 15 s, 3 × 20 s, 7 × 60 s, 17 × 180 s, and 6 × 600 s) true sinograms (ring difference 84) and reconstructed with a 2D filtered back-projection algorithm with corrections for detector geometry, efficiencies, attenuation, and scatter and dead time and were decay-corrected to the start of acquisition. Static images of the complete emission acquisition (0-120 min) were reconstructed with the manufacturer's proprietary iterative 3D algorithm (6 subsets and 4 iterations). SUVs were calculated by normalizing regional radioactivity for injected radioactivity and body weight.

### 2.4. Molecular Structure

Reverse-phase C18 and reverse-phase chiral HPLC analysis of [^18^F]SDM-4MP3 were performed as described (*vide supra*). LC-MS was performed on an Advion Expression L MS using atmospheric pressure chemical ionization (APCI). ^1^H and ^13^C NMR spectroscopy were carried out at the CSICOMP NMR Facility, University of Toronto, using either a Bruker Avance III 400 MHz or Agilent DD2 500 MHz NMR spectrometer.

## 3. Results and Discussion

Four healthy participants were recruited, of which two (70 F and 44 F) received [^18^F]SDM-4MP3 and two (50 F and 67 M) received [^18^F]SynVesT-1. Chronologically, the [^18^F]SDM-4MP3 human imaging with P1 occurred first, and the unexpected results triggered the imaging of the second pilot subject, P2, followed by the comparison experiments with [^18^F]SynVesT-1 precursor acquired from Yale or Pharmasynth. Retrospective investigations were undertaken using several methods to assess potential causes of the discrepancy in human imaging results. It was verified that the correct (*R*)-enantiomer was being used prior to human imaging scans, in light of a known loss of specific binding of [^18^F]SynVesT-1 with the (*S*)-enantiomer [12].

[^18^F]SDM-4MP3 was synthesized in radiochemical yields of 8.11 ± 2.03% (not decay corrected, starting from [^18^F]fluoride eluted to the reactor), radiochemical purity of >95% and molar activity of 224 ± 158 GBq/*μ*mol (*n* = 6). Radiochemical identity and enantiomeric excess (96%) were determined using authentic SynVesT-1 reference standard provided by Yale. [^18^F]SDM-4MP3 coeluted with SynVesT-1 on both reverse phase C18 HPLC and chiral HPLC.

**Figure 1:**
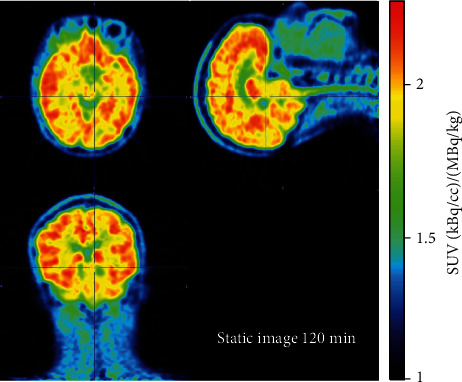
120-minute static image (SUV units) for the first participant (P1), injected with [^18^F]SDM-4MP3, scaled to maximum observed SUV.

**Figure 2:**
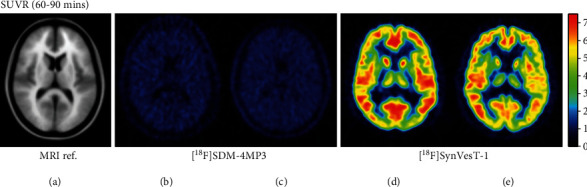
Average axial views in MNI space. (a) MRI shown for reference of SUVR, 60-90 min, centrum semiovale reference tissue, for subjects imaged with [^18^F]SDM-4MP3 (b, c), and [^18^F]SynVesT-1 (d, e).

A static image of the 120 min dynamic scan showing the average activity concentration for the first [^18^F]SDM-4MP3 recipient (P1) is shown in Figure 1. Although, as expected, there was greater average activity concentration in the synapse-rich gray matter than the white matter, the contrast between gray and white matter in this subject can be explained by differences in blood volume alone [21]. The large difference in SUVR is apparent in a side-by-side comparison of the SUVR images from [^18^F]SDM-4MP3 and [^18^F]SynVesT-1 (Figures 2(b) and 2(c) vs. Figures 2(d) and 2(e)), where SUVR for [^18^F]SDM-4MP3 was under 2 throughout the brain, and greater than 7 in cortical regions for [^18^F]SynVesT-1.

**Figure 3:**
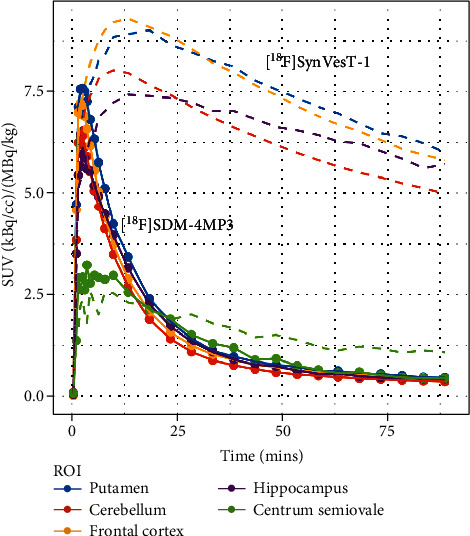
Time-activity curves (SUV units) for an example human subject for [^18^F]SDM-4MP3 (solid line) and [^18^F]SynVesT-1 (dotted line). Data is from P1 and P3.

Figure 3 shows the TACs for the frontal cortex, putamen, hippocampus, cerebellum, and centrum semiovale for a representative PET scan with [^18^F]SDM-4MP3 and [^18^F]SynVesT-1 in a participant (P1 and P3, respectively). Following injection of [^18^F]SDM-4MP3, despite high initial brain penetration (SUV of ~7) consistent with expectations, the peak occurred much earlier (2.5 min vs. 20 min), and the radiopharmaceutical showed rapid clearance from brain in sharp contrast to that based on previous human PET data observed (Figure 3; dotted line) and expected [8] for [^18^F]SynVesT-1. In fact, the [^18^F]SDM-4MP3 TACs appeared more like the blocking study data, suggesting that specific binding was minimal or absent. Nevertheless, the initial high peak activity concentration for [^18^F]SDM-4MP3 was evidence that the intended dose was delivered during injection and was able to reach the brain, albeit radiotracer binding to brain tissue vs. free in plasma was not determined.

Once it was confirmed that there were no issues with radiopharmaceutical injection and PET image processing, physiological differences were considered. It was noted that P1 (70 y.o. female) was significantly older than the subjects previously reported with [^18^F]SynVesT-1 PET scans in healthy controls (males, 44 ± 13 y) [8], and P1 was also on several medications (levothyroxine, rosuvastatin, and hydrochlorothiazide) that have unknown interactions with the radiopharmaceutical. This led to the scanning of a second participant to rule out subject-specific factors. To help rule out age effects, P2 was selected to be younger (age 44 y.o. vs. 70 y.o.; see Table 1). However, PET imaging results for P2 were similar to those of P1 (Figures 2(b) and 2(c)). Idiosyncratic biological differences such as a previously unknown genetic polymorphism affecting radiopharmaceutical binding (e.g., those observed for radiopharmaceuticals targeting the translocator protein 18 KDa [22, 23]) were considered to be less likely. We also evaluated the differences in our injection protocols, since we had used a slow manual bolus injection of 2-4 mL vs. a 1 min infusion from the previous publication [8]; however, these were not expected to cause a significant difference, particularly after the uptake peak.

**Figure 4:**
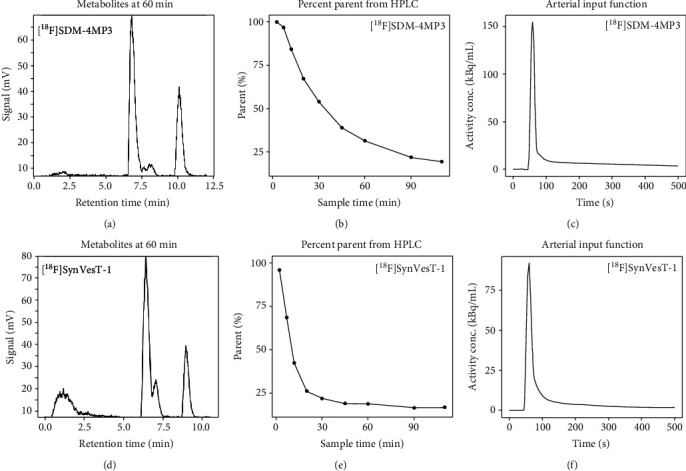
Example arterial blood results comparing [^18^F]SDM-4MP3 to [^18^F]SynVesT-1. (a) HPLC radiochromatogram from the 60 min discrete plasma sample, (b) parent fraction from all manual samples, (c) arterial plasma input function for [^18^F]SDM-4MP3. (d) HPLC radiochromatogram from the 60 min discrete plasma sample, (e) parent fraction from all manual samples, (f) arterial plasma input function for [^18^F]SynVesT-1.

Differences in metabolism for both radiopharmaceuticals in blood were subsequently assessed. Radiometabolite peaks for [^18^F]SynVesT-1were qualitatively very similar at both of our laboratories (not expected to be identical at CAMH and Yale due to differences in equipment and procedure, but the number of peaks and relative proportions were quantified). We also compared the radiometabolites between [^18^F]SDM-4MP3 and later scans that were conducted with [^18^F]SynVesT-1 (Figure 4), which had similar results. The rate of radiometabolism was slightly slower with [^18^F]SDM-4MP3 (Figures 4(b) and 4(e)). Minor differences were ascribed to participant metabolism and pressure differentials affecting the retention times in the column-switching HPLC.

The arterial input function was typical in appearance for [^18^F]SynVesT-1 [24] (Figures 4(c) and 4(f)). Overall, differences in blood were considered unlikely to account for brain uptake differences at this stage. To confirm this, kinetic quantification was performed using the arterial input functions generated for [^18^F]SDM-4MP3. A reasonable fit to the one-tissue compartment model was obtained, yielding *V*_*T*_ estimates that were more than a factor of 10 lower than expected (Table 2).

**Figure 5:**
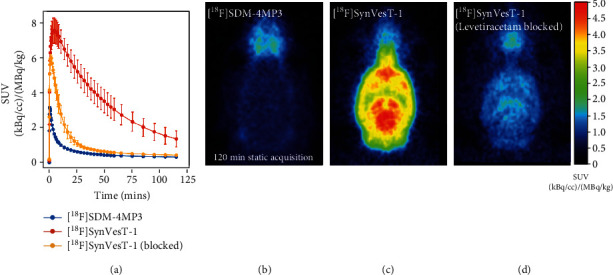
Preclinical comparison of [^18^F]SDM-4MP3 and [^18^F]SynVesT-1 in rats. (a) Whole-brain time activity curves from 0-120 min postinjection. Blocking study data from [^18^F]SynVesT-1 was acquired 15 min following injection of 30 mg/kg levetiracetam, summed 120 min images showing uptake of (b) [^18^F]SDM-4MP3, (c) [^18^F]SynVesT-1, and (d) [^18^F]SynVesT-1 under levetiracetam blocking condition.

Having ruled out human factors in imaging (differences between participants, errors in acquisition and analysis), a preclinical PET imaging investigation was carried out. A 0-120 min static image of [^18^F]SDM-4MP3 uptake in rat, using precursor from the commercial supplier, showed very limited brain uptake (Figure 5(b)), which was barely above tissues outside of the brain. In contrast, images from [^18^F]SynVesT-1 acquisition, using precursor obtained from Yale, showed high brain uptake with SUVs close to 8 (see Figure 5(c) for an example), consistent with previously reported results in rodents [15]. The TACs for both radiotracers are compared in Figure 5(a). To a greater extent than the human data, the initial peak uptake in the rodent brain was reduced from 7.6 ± 0.7 SUV for [^18^F]SynVesT-1 to 3.2 SUV for [^18^F]SDM-4MP3, and the whole-brain TACs showed much faster clearance for [^18^F]SDM-4MP3 vs. [^18^F]SynVesT-1 (Figure 5). It is noteworthy that the brain uptake of [^18^F]SDM-4MP3 was lower than [^18^F]SynVesT-1 scans performed under preblocking conditions with levetiracetam (Figures 5(a) and 5(d)). The preclinical observations led to the conclusion that the two precursor compounds had to be different and prompted an investigation into the structural characterization of the radiolabeling precursor obtained from the commercial vendor (ABX).

**Figure 6:**
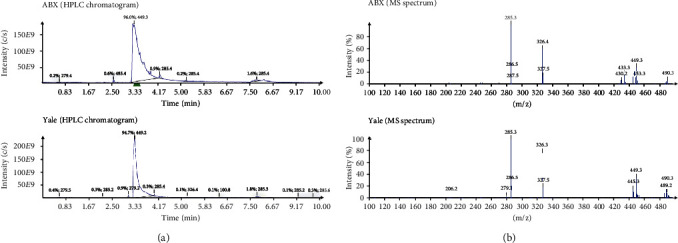
HPLC chromatograms (a) and mass spectrometry (MS) analysis (b) from precursors obtained from ABX and Yale. Precursor from an alternative commercial supplier (PharmaSynth) was also tested but was not appreciably different from the lot obtained from Yale (data not shown). The retention times for both samples were 3.30 min (ABX) and 3.31 min (Yale). The fragmentations seen in the mass spectra are similar, albeit with differing intensities, *m*/*z* 449.3 [M+H]^+^ and *m*/*z* 490.3 and the [M+CH_3_CN+H]^+^ signals are clearly detectable in both mass spectra.

**Figure 7:**
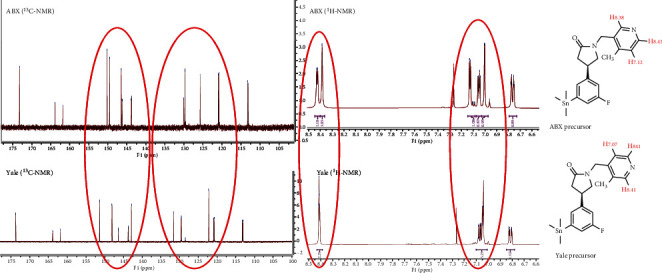
^13^C and ^1^H NMR spectra of precursors obtained from ABX and Yale (aromatic region shown with discrepancies outlined in red). Annotated protons for the pyridine ring for both precursors shown on the right. Precursor lot from PharmaSynth was also tested but was not appreciably different from the Yale lot (data not shown).

**Figure 8:**
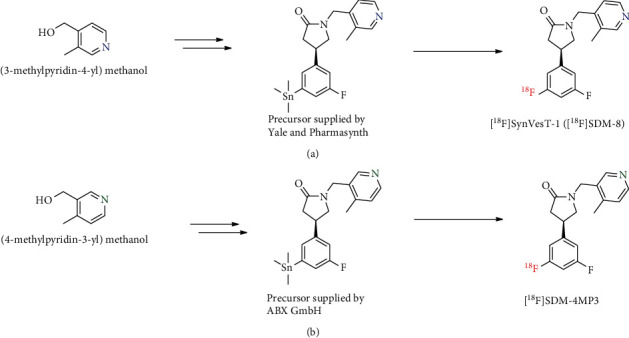
(a) Simplified synthesis route of the precursors by Yale and PharmaSynth and radiosynthesis of [^18^F]SynVesT-1 ([^18^F]SDM-8). Nitrogen in pyridinyl ring is shown in blue. (b) Simplified synthesis route of the precursor by ABX and radiosynthesis of [^18^F]SDM-4MP3. Differentiating pyridinyl nitrogen is shown in green. Reaction conditions for radiosynthesis: Cu(OTf)_2_, pyridine, [^18^F]KF, DMAc, 110°C, 20 min.

An investigation comparing the precursor from ABX with precursor lots synthesized at Yale and PharmaSynth AS using LC-MS showed that all three precursors had a similar retention time (3.30-3.32 min), and the same molecular mass of 448 g/mol (*m*/*z* 449.3 [M+H]^+^) with characteristic isotope patterns for tin and fluorine (Figure 6). The ^1^H- and ^13^C-NMR spectra showed clear differences (Figure 7). The ABX precursor differed from that of Yale and PharmaSynth in the aromatic region (notably between 155-140 ppm in ^13^C-NMR spectra and 8.5-8.3 and 7.2-7.0 ppm in ^1^H-NMR spectra). The peaks at 8.5-8.3 in the ^1^H-NMR spectra can be assigned to the orthoprotons on the pyridinyl ring. After analysis of chemical shifts and coupling constants of the aromatic protons, it was speculated that the substituents on the pyridinyl ring were different for the precursor provided by ABX compared to the precursors provided by Yale and PharmaSynth. A ^1^H-^1^H COSY NMR study confirmed that the ABX precursor was incorrectly characterized and was determined to have a 4-methyl-pyridinyl-3-yl moiety (SDM-4MP3) instead of the desired 3-methyl-pyridinyl-4-yl group (SynVesT-1). The radiopharmaceutical syntheses using both precursors to produce [^18^F]SynVesT-1 and [^18^F]SDM-4MP3 are shown in Figure 8.

It was later confirmed by ABX that the wrong material was delivered due to an incorrect reagent being used during the synthesis of the precursor material ((4-methylpyridin-3-yl)methanol was incorrectly used instead of (3-methylpyridin-4-yl)methanol). Previously published *in vitro* binding assays had revealed a loss of affinity to SV2A for SDM-4MP3 (SynVesT-1 *K*_*i*_ = 3.1 nM, SDM-4MP3 *K*_*i*_ = 291 nM), due to the reduced ability of the pyridine nitrogen to act as a hydrogen bond acceptor in this configuration [25, 26], which corroborated our *in vivo* imaging results. Although [^18^F]SDM-4MP3 is a structural derivative of [^18^F]SynVesT-1, this compound has dramatically reduced binding to SV2A (Figure 8). This phenomenon of fluorinated analogs of a molecule having dramatically different biological behaviour has been well documented in medicinal chemistry, often referred to as the “fluorine effect,” and has also been documented extensively in the radiopharmaceutical chemistry literature, for example with ring-fluorinated isomers of [^18^F]fluoro-L-DOPA [27–29].

## 4. Conclusions

Anomalous imaging results from an [^18^F]SynVesT-1 PET study spurred the discovery of incorrectly synthesized precursor material from a commercial vendor, resulting in an inadvertent first-in-human trial of the 4-methyl-pyridinyl analogue of [^18^F]SynVesT-1, namely, [^18^F]SDM-4MP3.

## Figures and Tables

**Table 1:** Participant information and injection parameters.

Participant	[^18^F]SDM-4MP3		[^18^F]SynVesT-1	
	P1	P2	P3	P4
Age (*y*)	70	44	50	67
Sex	F	F	F	M
Body weight (kg)	89.8	54	90	73.5
Smoking status	Nonsmoker	Non-smoker	Non-smoker	Non-smoker
Medications	Levothyroxine, rosuvastatin, hydrochlorothiazide	None	Amitryptyline	None
Injected dose (MBq)	189.00	196.36	182.68	185.87
Molar activity at injection (TBq/mmol)	272.8	138.9	221.3	99.1
Injected mass (*μ*g)	0.209	0.427	0.250	0.567

**Table 2:** *V*_*T*_ estimates from all four [^18^F]SDM-4MP3 and [^18^F]SynVesT-1 participants in selected ROIs. Values are *V*_*T*_ followed by SE (%) from fitting of 1-TCM.

ROI	[^18^F]SDM-4MP3 *V*_T_		[^18^F]SynVesT-1 *V*_T_	
	P1	P2	P3	P4
Putamen	2.0 (1.3%)	2.1 (3.4%)	16.1 (0.5%)	22.23 (4.7%)
Cerebellum	1.6 (1.4%)	1.8 (3.7(%)	13.1 (0.7%)	16.3 (4.9%)
Frontal cortex	1.8 (1.6%)	1.9 (3.4%)	15.4 (0.4%)	18.3 (5.1%)
Hippocampus	1.8 (1.4%)	1.8 (3.4%)	14.9 (0.6%)	17.0 (6.2%)
Centrum semiovale	1.6 (1.8%)	1.7 (2.0%)	2.9 (1.4%)	3.5 (7.7%)

## Data Availability

Supporting data is available upon request by contacting the corresponding authors.
